# ANSH: Multimodal Neuroimaging Database Including MR Spectroscopic Data From Each Continent to Advance Alzheimer’s Disease Research

**DOI:** 10.3389/fninf.2020.571039

**Published:** 2020-10-21

**Authors:** Pravat K. Mandal, Kanika Sandal, Deepika Shukla, Manjari Tripathi, Kuldeep Singh, Saurav Roy

**Affiliations:** ^1^NeuroImaging and NeuroSpectroscopy (NINS) Laboratory, National Brain Research Centre, Manesar, India; ^2^Florey Institute of Neuroscience and Mental Health, Parkville, VIC, Australia; ^3^Department of Neurology, All Indian Institute of Medical Sciences, New Delhi, India

**Keywords:** Alzheimer’s disease, diagnostic marker, neurochemical, glutathione, neuroimaging, behavioural database

## Abstract

Alzheimer’s disease (AD) is a devastating neurodegenerative disorder affecting millions of people worldwide. The etiology of AD is not known, and intense research involving multimodal neuroimaging data (e.g., MRI, functional MRI, PET etc.) is extensively used to identify the causal molecular process for AD. In this context, various imaging-based databases accessible to researchers globally, are useful for an independent analysis. Apart from MRI-based brain imaging data, the neurochemical data using magnetic resonance spectroscopy (MRS) provide early molecular processes before the structural or functional changes are manifested. The existing imaging-based databases in AD lack the integration of MRS modality and, thus, limits the availability of neurochemical information to the AD research community. This perspective is an initiative to bring attention to the development of the neuroimaging database, “ANSH,” that includes brain glutathione (GSH), gamma aminobutyric acid (GABA) levels, and other neurochemicals along with MRI-based information for AD, mild cognitive impairment (MCI), and healthy subjects. ANSH is supported by a JAVA-based workflow environment and python providing a simple, dynamic, and distributed platform with data security. The platform consists of two-tiered architecture for data collection and management further supporting quality control, report generation for analyzed data, and data backup with a dedicated storage system. The ANSH database aims to present a single neuroimaging data platform incorporating diverse data types from healthy control and patient groups to provide better insights pertaining to disease progression. This data management platform provides flexible data sharing across users with continuous project monitoring. The development of ANSH platform will facilitate collaborative research and multi-site data sharing across the globe.

## Introduction

**Figure 1:**
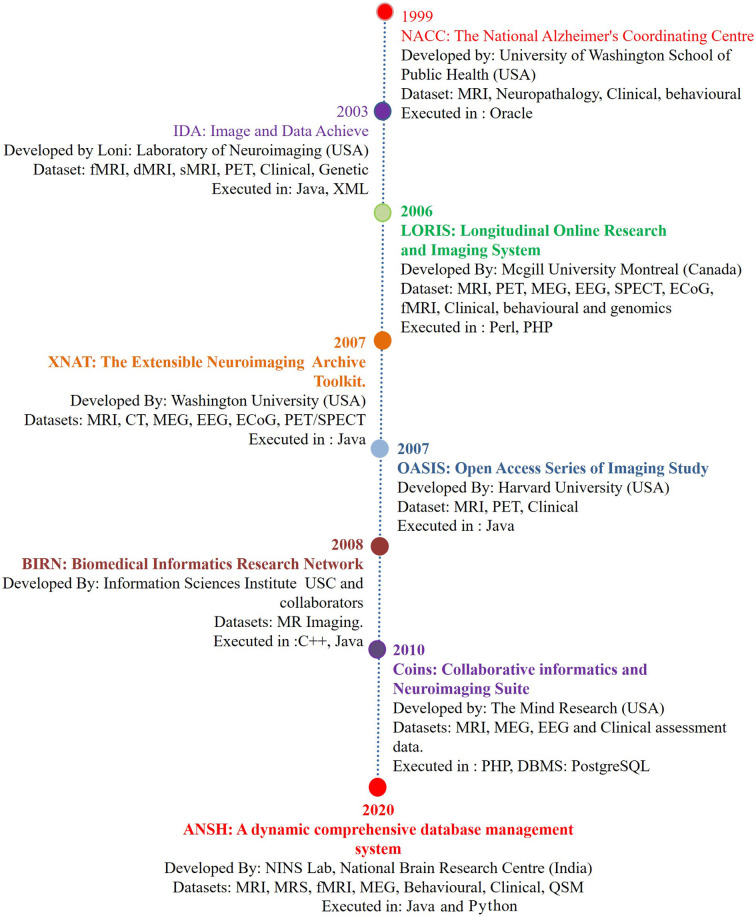
The chronological development of various neuroimage-based database pertaining to Alzheimer’s disease (AD) research with a variety of modalities. (1) National Alzheimer’s Coordinating Center (NACC; Cronin-Stubbs et al., 2000; Beekly et al., 2004). (2) IDA-LONI (Rex et al., 2003; Neu et al., 2005). (3) Longitudinal Online Research Imaging System (LORIS; Das et al., 2011). (4) Neuroimaging Archive Toolkit (XNAT; Marcus et al., 2007). (5) Open Access Series of Imaging Study (OASIS; Marcus et al., 2007). (6) Biomedical Informatics Research Network (BIRN; Keator et al., 2008). (7) Collaborative Informatics and Neuroimaging Suite (COINS; Scott et al., 2011). (8) ANSH: a dynamic comprehensive database management system. Abbreviations: DTI, diffusion tensor imaging; ECoG, electrocorticography; EEG, electroencephalogram; fMRI, functional magnetic resonance imaging; MEG, magnetoencephalography; MRS, magnetic resonance spectroscopy; PET, positron emission tomography; QSM, quantitative susceptibility mapping; SPECT, single-photon emission computerized tomography. MRS data specifically critical antioxidant, receptors, and brain energy metabolites will be available through the ANSH.

Alzheimer’s disease (AD) is a major neurodegenerative disorder, and the number of AD patients is increasing globally with each passing year, and disease-modifying treatment is not available. Although pathophysiologic knowledge of AD from existing hypotheses like amyloid beta deposition (Hardy and Higgins, 1992) has helped immensely to understand the disease process, the causal process for AD has not been identified. Neuroimaging modalities involving MRI, fMRI, PET, and behavioral studies have provided the associated structural and behavioral changes in the disease process (Gorgolewski et al., 2016). The first such database was reported by the National Alzheimer’s Coordinating Center (NACC), which mainly involved MRI, and the genetic and behavioral dataset of healthy old (HO), and AD patients (Cronin-Stubbs et al., 2000; Beekly et al., 2004). These databases created an opportune situation for the sharing of imaging-based data with researchers. There are other databases from Image Data Archive, Laboratory of NeuroImaging (IDA LONI; Rex et al., 2003; Neu et al., 2005), Longitudinal Online Research Imaging System (LORIS; Das et al., 2011), Extensible Neuroimaging Archive Toolkit (XNAT; Marcus et al., 2007), Open Access Series of Imaging Study (OASIS; Marcus et al., 2007), Biomedical Informatics Research Network (BIRN; Keator et al., 2008), and Collaborative Informatics and Neuroimaging Suite (COINS; Scott et al., 2011). The list of databases is expanding, and only a few specific features mainly structural and functional related to AD are presented in Figure 1. Subsequently, neurochemical data is added in the present dataset “ANSH” to bridge the gap (Figure 1). MR spectroscopy (MRS) is a potent non-invasive modality to identify the various neurochemicals involved in the early disease process. MRS-driven outcomes provide information that is critically involved in the transition of normal healthy person to mild cognitive impairment (MCI).

Various neurochemicals [e.g., *N*-acetyl aspartate (NAA), myo-Inositol (mI), creatine (Cr), choline (Cho), etc.; Doraiswamy et al., 1998; Mandal et al., 2015], neurotransmitter [e.g., gamma aminobutyric acid (GABA), glutamate, glutamine, etc.; Bai et al., 2015), antioxidant, and glutathione (GSH) level (Mandal et al., 2012, 2015; Shukla et al., 2020] can be quantified in AD brain using MRS. The NAA/mI ratio and the GSH levels from the hippocampus are correlated to cognitive decline in various behavioral studies (Doraiswamy et al., 1998; Mandal et al., 2015). In MRS studies, the depletion of GSH in the hippocampus, frontal cortices, and anterior cingulate cortices (Mandal et al., 2012, 2015; Shukla et al., 2020) has been validated from various independent postmortem studies (Gu et al., 1998; Sultana et al., 2008; Ansari and Scheff, 2010).

Hence, the inclusion of neurochemical data is required and will play a profound role in AD research for identifying a causal molecular process for AD, possible therapeutic development, and monitoring disease progression. ANSH is the first platform where antioxidant, neurotransmitter, and energy metabolites are discussed and will be available to the research community.

## A Chronological Development of Various AD-Based Database

Database and associated data-processing platforms are powerful tools for supporting medical data mining and discovery from the wealth of routinely acquired clinical and imaging data. This facilitates better information, individualized and optimized patient care (Bui et al., 2013).

Since then, many initiatives progressed toward the open sharing and reusability of the original data. This section briefly describes more details of these databases. The University of Washington School of Public Health supported by the National Institute of Health (NIH) started the Alzheimer’s Disease Research Center (ADRC) with a mandate to provide a comprehensive advanced AD research and related disorders from 39 ADRCs at various medical schools across the United States. NACC started with a behavioral and genetic dataset platform and then gradually incorporated MR imaging (Cronin-Stubbs et al., 2000; Beekly et al., 2004). Subsequently, an increasing number of experiments led to the generation of heterogeneous datasets, with an urgent need for standardization and distribution of this information. This initiative resulted in the creation of IDA-LONI for data sharing concerning the disease progression from various research sites globally^1^. IDA-LONI is a hub comprising approximately 138 studies from various disease datasets with new studies added overtime (Petersen et al., 2010). LONI is fortified with upload, download, quality check (QC), processing, and various other user-level sharing features. The Alzheimer’s disease neuroimaging initiative (ADNI; Petersen et al., 2010) and the human connectome projects are associated with this platform (Rex et al., 2003). LORIS is a web-based platform for neuroimaging studies (Das et al., 2011). LORIS consists of a wide range of datasets including neurological, behavioral, and imaging data from anatomical, functional maps, atlases, and MRI models. LORIS streamlined a framework for storing and processing behavioral, clinical, neuroimaging, and genetic data. The combination of the software platform and web-based approach for data management, throughput task, and data sharing to the approved users was supported by the Neuroimaging Archive Toolkit (XNAT; Marcus et al., 2007), Analysis of Functional Neuroimages (AFNI; Cox, 1996), Human Imaging Database (HID; Marcus et al., 2011), and Brain Imaging Data Structure (BIDS; Gorgolewski et al., 2016), which provide the user with the data management tools for a better analysis of data across a diverse number of neuroimaging datasets. The Neuro-Imaging Tools and Resource Collaboratory (NITRC) has played a vital role in hosting all the neuroimaging software repository, data, and toolboxes under one platform^2^.

OASIS (Marcus et al., 2007) is a dedicated project involving brain MRI and PET longitudinal data available to the scientific community. The BIRN package offers an amalgamated and distributed infrastructure for the storage, retrieval, analysis, and documentation of biomedical imaging datasets (Keator et al., 2008). BIRN uses XNAT and HID for data acquisition and management (Keator et al., 2008). In progression, COINS is comprised of 300 studies consisting of 19,000 MRI, magnetoencephalography (MEG), and electroencephalogram (EEG) scans with more than 180,000 clinical assessments (Bockholt et al., 2010). The COINS database provides an optimized platform for data mining from multiorganization sources shared with added security and data tracking portal with Public Health Information (PHI; Bockholt et al., 2010).

## Importance of MR Spectroscopy Data and Application

**Figure 2:**
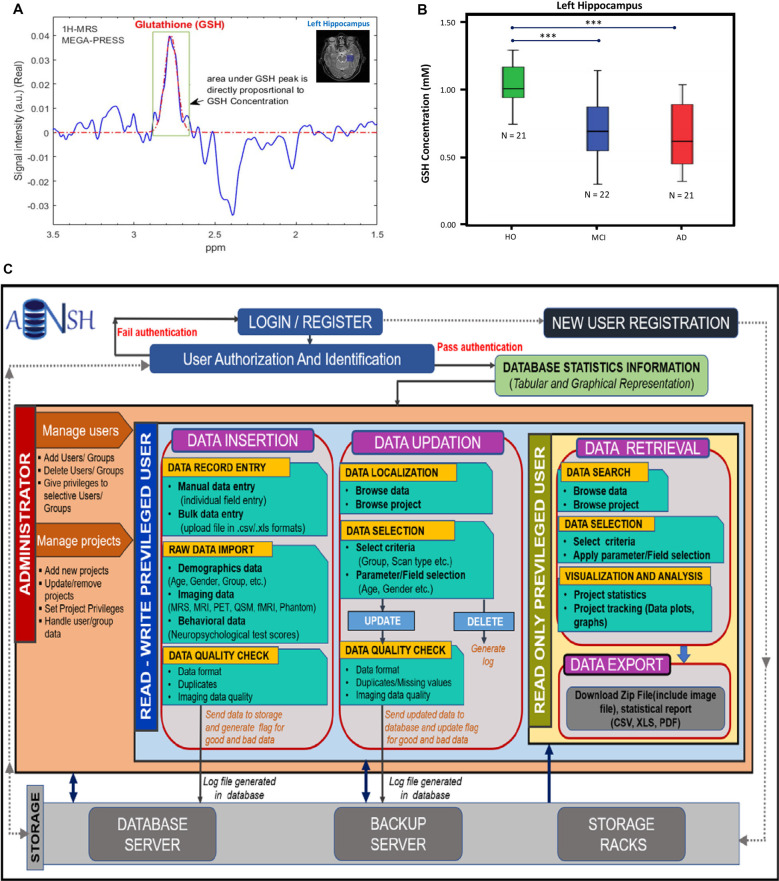
**(A)** 1H-MRS MEGA-PRESS edited spectra for *in vivo* glutathione (GSH) estimation in the left hippocampus of a HO brain. **(B)** Box–whisker plot showing significant GSH concentration depletion in the mild cognitive impairment (MCI) and AD groups compared to the HO group. ***The significance level was set at *p* < 0.001 (Mandal et al., 2015). **(C)** This workflow illustrates the conventions for the construction of database and management of data. The access of data is provided by the user authentication. The privileged user is provided with two main segments, i.e., data insertion and updation, where the user can review data and can make appropriate changes. Both segments follow the raw image quality control (QC) before putting the data in the backup servers. It will also keep a track of the data usage and generate logs for the changes. Read-only privileged users are enriched with the segment for data retrieval to export image data and the data reports using various search filters and data selection parameters. All segments are interconnected by the backend server and the rack system of the ANSH.

MRS data can be generated from any part of the brain using single-voxel mode or multivoxel mode (Mandal, 2007). MRS data is generally smaller in size and easy to handle compared to MRI-based data. Various advanced packages are available to process MRS data, and absolute quantitation of various neurochemicals is also possible (Mandal and Shukla, 2020). These MRS-processing packages can be added with a suitable plugin so that processed MRS data can be utilized in multimodel big data analytics (Sharma et al., 2019). Various MRS pulse sequences are now available to detect specific neurochemicals (GABA, GSH, glutamate/glutamine) without any ambiguity (Terpstra et al., 2003). Figure 2A represents the MRS spectra from the left hippocampus by placing the single-voxel (25 × 25 × 25 mm^3^), MRS data was acquired using a 3T MRI (Achieva, Philips) scanner, and processed using the KALPANA software (Mandal and Shukla, 2020). Figure 2B represents absolute quantitation of GSH in relevant amount (mM) from the left hippocampus of HO, MCI, and AD patients (Mandal et al., 2015). Significant depletion of GSH level from the left hippocampus, as detected by *in vivo* MRS, is sensitive for comparing HO, MCI, and AD patients. MRS is also the only technique that can be used to detect the GSH conformers *in vivo*, and these conformers are likely to play an important role in the AD disease process (Mandal et al., 2017, 2019; Shukla et al., 2020). The changes in the GSH level are susceptible to AD pathology only. Data indicated that, GSH level in the left cerebellum of AD and HO did not alter significantly (*p* = 0.536); however, the specific change in GSH in the hippocampal regions in the same AD and HO groups was found to be significant (*p* < 0.001; Mandal et al., 2015). The database involving ^31^P MRS is critical to understand the impaired energy metabolism process (e.g., increased hippocampal pH) in the AD brain in contrast to the age-matched normal brain (Mandal et al., 2012; Rijpma et al., 2018).

This perspective will bridge the gap providing the unique neurochemical data in AD research.

## Technical Details of the ANSH Database in Brief

### Key Features

ANSH, a dynamic and distributed data management platform, follows a two-tired architecture that includes a graphical user interface, processing pipeline, and database server. The ANSH supports a JAVA-based workflow environment and python for storage with flexible data access and data sharing among users. In addition, QC ensures an improved database management. The ANSH also provides a report generation feature with the additional functionality of continuous project monitoring using data visualization and statistical analysis.

Some key features of the ANSH are: (1) distributive platform; (2) user login with privacy implementations; (3) quality check; (4) image viewer; (5) centralized approach to fetch data; (6) real-time tracking and backup of the database; (7) heterogeneous data; (8) effortless import/export of data with the use of data-processing pipelines; and (9) report generation.

### Data Types and Quality Control

ANSH provides a dynamic and comprehensive database management system for the heterogeneous neuroimaging datasets, specifically MRI and multinuclear MRS for HO, MCI, and AD categories along with the neuropsychological test scores. The MRI data consist of diverse 3D T1, T2-weighted, Flair, and QSM images for different age groups of healthy young as well as HO subjects. Other imaging modalities such as PET, fMRI, and DTI will also be added to the ANSH database. MRS data for GSH and GABA are provided for age-matched HO, MCI, and AD groups along with T1, T2-weighted, and Flair MRI dataset. The neuropsychological score broadly constitutes from the mini-mental state examination, clock drawing test, trail making tests. The flow diagram of the ANSH is presented in Figure 2C.

### ANSH User-Flow Schematic

The ANSH incorporates three levels of users including administrator, write/read privileged, and read privileged only. The administrator is the top-level user, responsible for assigning other user’s rights as well as creating and managing the new and existing project information. The administrator only holds the right to completely remove the specific project data from the database. Users with the read and write privileges can read and write the specific data according to the permission given to them by the administrator, whereas the user with the read privileges will only be able to read a selective database for the specific projects as permitted by the administrator. Each user can export the selected data from the specified project.

### Dataset Handling

Data entry in the ANSH database can be accomplished individually using data entry forms or imported in bulk. Data insertion and updation rights are held by the administrator and given to the write-privileged users for the specified projects. Data retrieval rights are entitled to all users. The ANSH also provides detailed data report, data visualization plots (i.e., bar and pie charts) as image files and statistical information in the form of pdf and excel which can be exported afterwards.

### Data Processing, Storage, and Security

In the ANSH database management system, the user can access the database through a secure desktop application. Data imported or entered, undergoes a rigorous quality assurance process with a quality flag level. This quality tagged data is sent to the server for storage and subsequently history logs are maintained and stored in the ANSH server tracking associated changes.

Data security is provided in multiple steps: (1) authorized users will be granted access to the database, (2) quarantine of sensitive files (e.g., user password file), where files are encrypted and hashed, (3) tracking user behavior against data, (4) successful/failed attempts to establish connection are logged to track intruders, (5) restricting user access by designing and granting appropriate user with limited administrative privileges, and (6) database backups are taken as a part of security protocol, where these backups allow to recover the lost data that may have resulted from hardware failure, data corruption, theft, or natural disasters.

## Conclusion and Future Directions

The ANSH database construction is a sincere attempt to bring the critical neurochemical information from HO, MCI, and AD patients to the global researchers for comparative analysis. WE will also add Parkinson disease data and other mental disorders data in ANSH database. This novel program is in an expanding stage and needs further infrastructure support to build a robust system to cater to the Indian and global brain research community.

## Data Availability Statement

The original contributions presented in the study are included in the article, further inquiries can be directed to PM through email pravat.mandal@gmail.com.

## Author Contributions

PM and DS conceptualized the idea and were involved in the manuscript writing and figure design. KSa was involved in the database design, manuscript writing, analysis of various databases and preparation of Figure 1. MT was involved in discussion as a clinical collaborator. KSi was involved in programming of the database development and expansion and preparation of Figure 2. SR was involved in Web-based application and front-end generation.

## Conflict of Interest

The authors declare that the research was conducted in the absence of any commercial or financial relationships that could be construed as a potential conflict of interest.

## Footnotes

^1^https://ida.loni.usc.edu

^2^https://www.nitrc.org
